# Supplementary figures and images for: Opposite polarity programs regulate asymmetric subsidiary cell divisions in grasses
Source: eLife. 2022 Dec 20;11:e79913. doi: 10.7554/eLife.79913 (PMC9767456; doi:10.7554/eLife.79913)

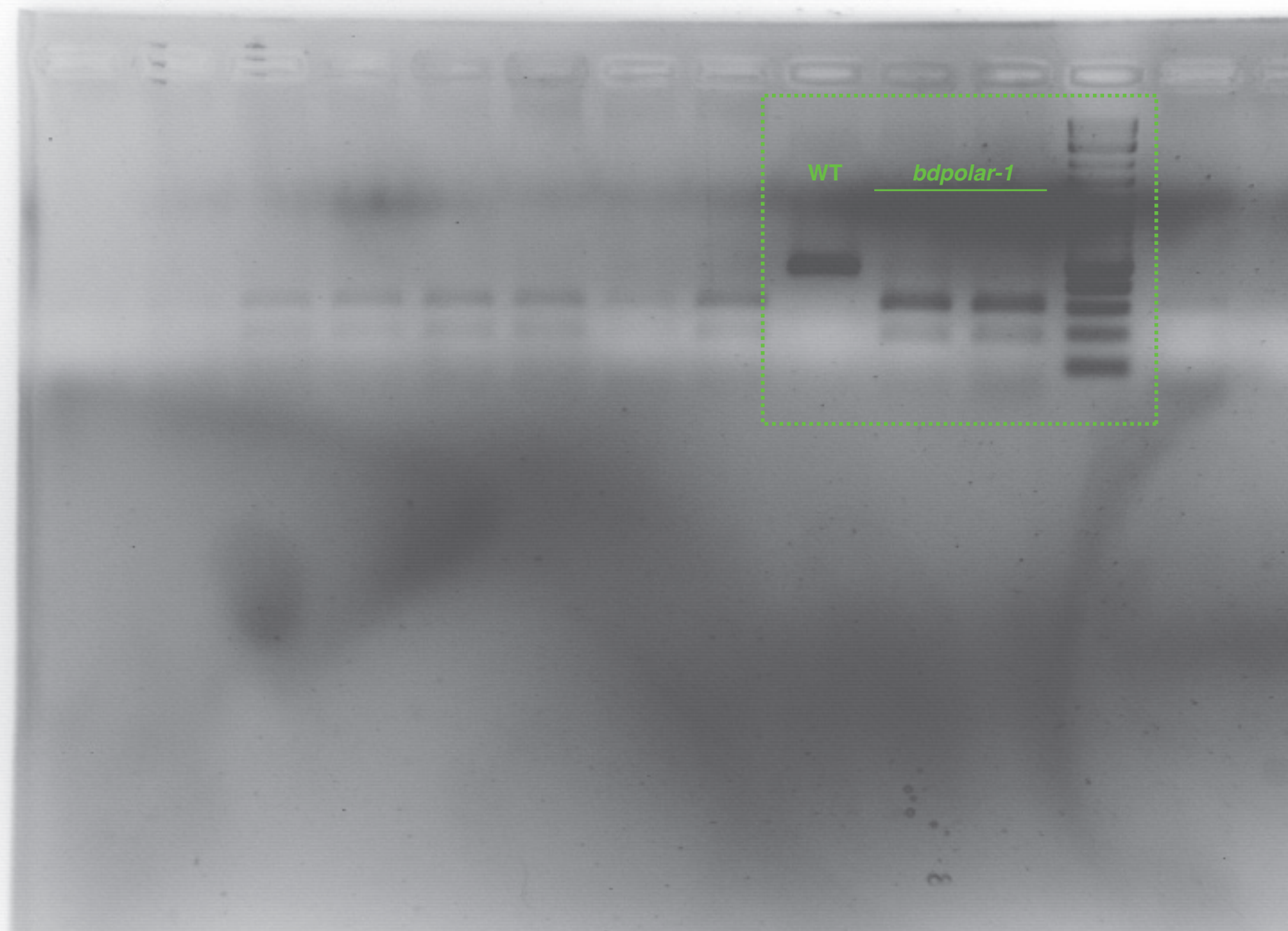

Supplement: Figure 1—figure supplement 2—source data 1. [file elife-79913-fig1-figsupp2-data1.zip › Source Data/FigureS1 - source data.pdf]

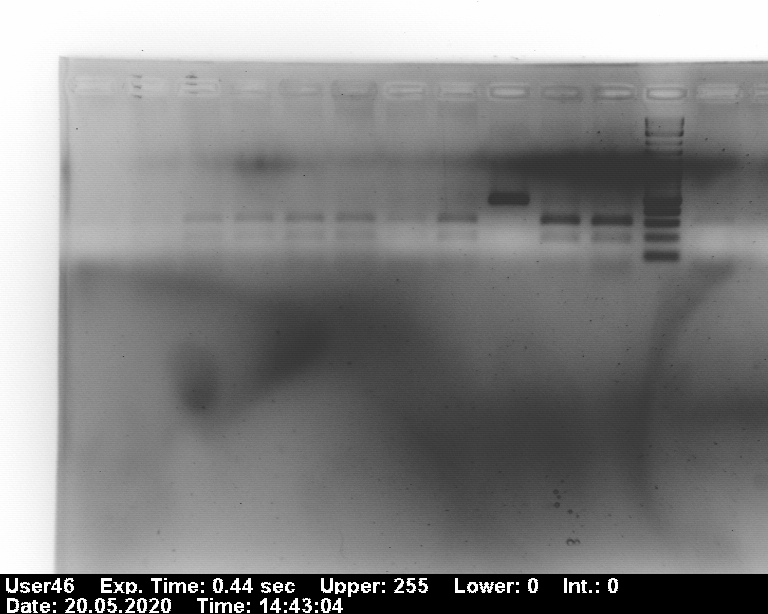

Supplement: Figure 1—figure supplement 2—source data 1. [file elife-79913-fig1-figsupp2-data1.zip › Source Data/FigureS1 - source data.tif]
